# Inhibitory effects of NaF on mitochondrial energy generation in human platelets *in vitro*

**DOI:** 10.3389/ftox.2024.1421184

**Published:** 2024-09-05

**Authors:** Tetsuhiro Tsujino, Tomoni Kasahara, Hideo Kawabata, Taisuke Watanabe, Koji Nishiyama, Yutaka Kitamura, Takao Watanabe, Hajime Okudera, Tomoharu Mochizuki, Takashi Ushiki, Tomoyuki Kawase

**Affiliations:** ^1^ R&D Division, Tokyo Plastic Dental Society, Tokyo, Japan; ^2^ Department of Orthopaedic Surgery, Niigata University Graduate School of Medical and Dental Sciences, Niigata, Japan; ^3^ Division of Hematology and Oncology, Graduate School of Health Sciences, Niigata University, Niigata, Japan; ^4^ Department of Transfusion Medicine, Cell Therapy and Regenerative Medicine, Niigata University Medical and Dental Hospital, Niigata, Japan; ^5^ Department of Hematology, Endocrinology and Metabolism, Faculty of Medicine, Niigata University, Niigata, Japan; ^6^ Division of Oral Bioengineering, Niigata University Graduate School of Medical and Dental Sciences, Niigata, Japan

**Keywords:** fluoride, platelets, aggregation, adhesion, respiration, ATP

## Abstract

**Background:**

fluoride is a beneficial ion that has been used in various fields, from industrial products to therapeutics. However, due to its narrow therapeutic index, fluoride sometimes acts as a toxic agent at relatively higher concentrations in the human body. Based on the interest in genetic stability, its cytotoxic effects have been investigated mainly in nucleated, adherent cells, such as fibroblasts. However, the sensitivity of blood cells, especially anucleate platelets, to fluoride is poorly understood. To fill this gap in the literature, we investigated the effects of relatively low levels of fluoride on platelet energy metabolism, function, and viability.

**Methods:**

Platelet-rich plasma (PRP) was prepared from 15 non-smoking healthy male adults (age: 28–63) and treated with NaF (0.5 or 1.0 mM) in microtubes for up to 3 days. Platelet function was evaluated based on aggregation and adhesion activities. Platelet energy metabolism was evaluated based on intracellular ATP levels, extracellular lactate levels, and respiration activities. The mitochondrial membrane potential (Em) and localization of reactive oxygen species (ROS) were visualized using cytochemical methods. Platelet viability was evaluated by cell counting and tetrazolium reduction.

**Result:**

NaF (1 mM) significantly reduced platelet viability and inhibited functions. Behind these phenomena, NaF substantially decreased mitochondrial Em and increased ROS production along with significant decreases in oxygen consumption and ATP levels. Simultaneously, NaF increased the lactate levels. Although not statistically significant, similar effects were observed at 0.5 mM NaF.

**Conclusion:**

At relatively low levels, NaF has the potential to attenuate platelet function probably primarily through the inhibition of mitochondrial energy generation. Cytotoxicity may be directly related to ROS production. These findings suggest that when used topically, for example, for caries prevention in the oral cavity, NaF could interfere with wound healing and tissue regeneration by endogenous and exogenously added platelets in the form of PRP.

## 1 Introduction

Fluoride is a versatile ion used in our lifestyle. Fluoride has been widely applied not only in industrial products but also in the medical field to increase the mechanical and chemical strength of bones and teeth. This application is based on evidence of the chemical reaction that fluoride can increase the acid resistance of hydroxyapatite, which is the main inorganic component of bone, enamel, and dentin, by replacing the hydroxyl group during the formation of the apatite crystal lattice (Aoba, 1997).

It should be noted that relatively higher concentrations (≈1.2 M) of fluoride compounds, mainly in the form of sodium fluoride (NaF), are topically applied to prevent dental caries and treat dentin hypersensitivity in the field of dentistry (Petersson, 2013; Carey, 2014; Vasisth et al., 2024). It has been popular and frequently used also adult fluoride varnish (Piesiak-Pańczyszyn et al., 2023). Thus, it is presumable that such higher levels of fluoride, even though the contact time is limited, may influence not only the oral mucosa but also wounds and inflammation in the oral cavity. Nevertheless, from a general toxicological point of view, the toxic effects of fluoride have been investigated mainly in cultured fibroblasts and bone cells, particularly in earlier studies (Helgeland and Leirskar, 1976; Holland, 1979; 1980). Although sensitivity to fluoride depends on the cell type and pH of the medium, fluoride provided by various compounds appeared to induce similar cytotoxic effects. Thereafter, the toxicological study of fluoride has been expanded to various adherent cell types and gradually clarified the molecular mechanism of cytotoxic action: fluoride inhibits protein synthesis, mitochondrial function, and ATP generation behind cell death and growth arrest (Jeng et al., 1998; Zhang et al., 2008; Liu et al., 2018). The review article very recently published explained the mechanism in more detail, especially the impact on mitochondria (Wei et al., 2022). In contrast, the sensitivity of non-adherent blood cells, such as platelets, to fluoride has rarely been investigated for cytotoxicity or functional activities.

Furthermore, the concept regarding the primary site of fluoride action, i.e., mitochondria, seems in conflict with the conventional application of fluoride in laboratory testing. NaF has been used as an anticoagulant and antiglycolytic agent and is routinely used at a concentration of 2.5 mg/mL (60 mM) or higher when used alone to test blood glucose levels (Chan et al., 1989; Chaiyasut et al., 2018). The anti-glycolytic effect is based on the concept that fluoride reduces glycolysis activity by inhibiting enolase, the key enzyme involved in the glycolysis pathway (Cimasoni, 1971; Qin et al., 2006). However, this concept originated from the early studies investigating fluoride’s effects on bacteria that depend solely on glycolysis for ATP generation (Hamilton, 1977; Kashket and Bunick, 1978). Coincidently, like bacteria, the major blood cells in quantity, erythrocytes, lack the mitochondrial ATP generation system (Mortensen et al., 2010). Thus, NaF can be anticipated to sufficiently preserve blood glucose levels in collected blood samples, even if NaF alone does not quickly inhibit glycolysis after blood collection (Ridefelt et al., 2014). Although it is practically acceptable in laboratory testing, fundamental questions about other blood cells remain to be clarified.

Although known as a chronic poison, fluoride cytotoxicity has been investigated mainly assuming the case of acute fluorosis that is caused by exposure to relatively high concentrations of fluoride. Thus, it can be speculated, but is not yet clearly demonstrated, how fluoride influences platelet function and viability (Fantl, 1968; Mürer and Stewart, 1977; Mürer et al., 1981; Kienast et al., 1987; Kondo et al., 1991). In the oral cavity, superficial wounds in the mucosal tissue heal rapidly. However, when surgical operation is performed to cure periodontitis, the wound may not easily be healed depending on the severity. In this case, there is a possible risk that platelets endogenously mobilized and accumulated around the injured site or exogenously provided as a form of PRP could be exposed to the relatively high concentrations of fluoride that are used for caries prevention in clinical settings. Concerning the mechanism of action, in addition, if fluoride acts on the glycolysis in platelets as in bacteria, dysfunction and reduced viability of platelets could be primarily due to the inhibition of glycolysis. Although platelets are thought to preferentially use glycolysis for ATP generation (Aibibula et al., 2018; Melchinger et al., 2019), it has not yet been demonstrated clearly how much the glycolysis is involved in the inhibitory action of fluoride in platelets. Platelets are the second most abundant blood cells and play important roles in hemostasis, wound healing, and immune responses (Nurden et al., 2008; Mezger et al., 2019; Gupta et al., 2020). To investigate how fluoride influences platelet fate and function, we examined the effects of NaF at relatively low levels (≤1 mM) on platelet viability, energy metabolism from glycolysis to oxidative phosphorylation (OXPHOS), and its functions *in vitro*.

## 2 Materials and methods

### 2.1 Preparation of PRP

The study design and consent forms for all procedures (project identification code: 2019-0423) were approved by the Ethics Committee for Human Participants at Niigata University (Niigata, Japan) and complied with the Helsinki Declaration of 1964, as revised in 2013. Informed consent was obtained from all subjects involved in the study.

Blood samples were collected from non-smoking healthy male volunteers (n = 15, age: 28–63 years). Peripheral blood was collected in glass vacuum blood collection tubes (Vacutainer^®^; BD Biosciences, Franklin Lakes, NJ, United States) containing 1.5 mL A-formulation of acid-citrate-dextrose solution (ACD-A) (Terumo, Tokyo, Japan). Donors positive for HIV, HBV, HCV, or syphilis antibodies were excluded. After centrifugation, blood samples with optically recognized hemolysis or chyle plasma were also excluded.

Pure-platelet-rich plasma (P-PRP) was prepared by horizontal centrifugation (415 g, 10 min). The upper plasma fraction (P-PRP) was transferred into 2 mL sample tubes and treated with NaF for 1–3 days by intermittent stirring with a tube roller mixer at ambient temperature (20°C–23°C) as described previously (Uematsu et al., 2023).

### 2.2 Platelet viability test

Platelet viability was evaluated by cell counting and conversion of formazan to tetrazolium salt. Platelets in platelet-poor plasma (PPP) were directly counted using an automated hematology analyzer (pocHiV-diff, Sysmex Corporation, Kobe, Japan). For the formazan converting capacity, after centrifugation (664 × g, 3 min), platelets were suspended in 0.5 mL of PBS and mixed with 30 μL of WST-8, which was provided as a component of Cell Counting Kit-8 (Dojindo, Kumamoto, Japan). After incubation at room temperature for 60 min, the platelet suspensions were centrifuged and the supernatants were subjected to spectrophotometric determination at 450 nm. The number of samples tested was 15.

### 2.3 Lactate measurement in extracellular plasma

Lactate levels in extracellular plasma were determined using a lactate meter that employs specific test strips based on the lactate oxidase enzyme electrode method (Lactate Pro2; Arkray, Kyoto, Japan) (Crotty et al., 2021). Because the lactate levels in stored whole blood samples are largely dependent on the glycolysis of red blood cells, its time-course changes in the form of PRP, which excludes red blood cells, were monitored to determine the levels of lactate released by platelets. The number of samples tested was 15.

### 2.4 Examination of platelet morphology using scanning electron microscopy

At the end of treatment, the platelets were filtered using a polycarbonate membrane (Nano percolator; Nisshin-EM, Tokyo, Japan), fixed with 2.5% neutralized glutaraldehyde, serially dehydrated in ethanol and t-butanol solutions, and freeze-dried (Toyoda et al., 2018).

For microscopic examination, the prepared samples were sputter-coated with a thin layer of gold to enhance the electrical conductivity, and subsequently examined under a high-resolution scanning electron microscope (TM-1000; Hitachi, Tokyo, Japan) operating at an acceleration voltage of 15 kV (Nakamura et al., 2023). This experiment was repeated thrice.

### 2.5 Visualization of reactive oxygen species (ROS) and mitochondria membrane potential (Em)

At the end of the treatment, platelets were immobilized on glass slides using a Cytospin 4 cytocentrifuge (Thermo Fisher Scientific, Waltham, MA, United States) and fixed with 10% neutral-buffered formalin. Total reactive oxygen species (ROS) were visualized with photo-oxidation resistant DCFH-DA dye (R253 ROS assay kit; Dojindo) and examined using a fluorescence microscope equipped with an excitation filter (465–495 nm) and a barrier filter (515–555 nm) (Eclipse 80i; Nikon, Tokyo, Japan). Alternatively, to evaluate mitochondrial membrane potential (Em), fixed platelets were stained using the MT-1 MitoMP Detection Kit (Dojindo) and examined using a fluorescence microscope equipped with an excitation filter (510–560 nm) and a barrier filter (590 nm) (Ushiki et al., 2022). It should be noted that this innovative dye can also be used for fixed cells (Laboratories, 2024). These experiments were repeated thrice.

### 2.6 Platelet aggregation assay

Platelet aggregation was examined by aggregometry as previously described (Uematsu et al., 2023). Briefly, at the end of treatment, platelet counts in P-PRP were adjusted to 20–40 × 10^4^/μL with PPP using the automated hematology analyzer (Sysmex Corporation), treated with 0.03% CaCl_2_, and activated with ADP (3 μM) (FUJIFILM Wako Pure Chemical Corp., Osaka, Japan) or collagen (2 μg/mL) (MCM collagen H; DS Medical Co. Ltd., Tokyo, Japan). Aggregation was monitored for 5 min using a spectrophotometer (PRP3000S; TAIYO, Osaka, Japan). The level of maximal aggregation was determined. The number of samples tested was 15.

### 2.7 Platelet adhesion assay

At the end of the treatment, platelets in 200 µL of P-PRP were stimulated with 0.2 μM ADP (FUJIFILM Wako Pure Chemical Corp.) and incubated for 10 min at room temperature to test platelet adhesion ability. In the second half (5 min) of this incubation period, 100 µL of glass microbeads (BZ-04, φ0.350–0.500 mm) (AS ONE, Tokyo, Japan) was added to the P-PRP samples. At the end of the incubation, the number of platelets in the supernatant was counted (Uematsu et al., 2023). The number of samples tested was 15.

### 2.8 Determination of platelet ATP

At the end of the treatment, P-PRP samples were centrifuged (664 × g, 4 min) and suspended in PBS. Platelets were counted and platelet ATP levels were determined with a luminescence ATP assay kit (Dojindo) using luminescence kit (AB-2200, Atto Corp., Tokyo, Japan). Data were normalized to platelet counts (Uematsu et al., 2023). The number of samples tested was 15.

### 2.9 Determination of platelet oxygen consumption (respiration)

Among the 15 male donors, the donors with chylomicronemia were excluded because triglyceride at higher levels seems to reduce the permeability of oxygen through the diagram over the oxygen electrode. Thus, 11 donors (28–63 years) were selected for this experiment. Oxygen consumption was measured using a Mitocell Respirometry System, which comprises a 782 oxygen meter and an RC300 respiration cell with a 1302 electrode (Strathkelvin Instruments Limited, North Lanarkshire, Scotland, United Kingdom).

According to the manufacturer’s instruction, the oxygen electrode was calibrated with air-saturated PBS (Calibration “high”) and 0.01 M disodium tetraborate solution containing a small amount of sodium sulfite (Calibration “zero”). The calibration was repeated for each measurement and the inner wall of the cell was cleaned by Kim Wipe to prevent the possible carryover of oxygen contents in the saturated PBS and the samples.

For sample preparation, P-PRP fractions were prepared as described above and PPP fractions were prepared by further centrifugation to exclude cell components. P-PRP and PPP fractions were fully added to 0.5 mL microtubes to minimize air space and air bubble formation and gently stirred intermittently for 24 h at room temperature. At the end of the incubation, platelets were excluded from the P-PRP samples to prevent unexpected adhesion of platelet and large molecules to the polyethylene membrane covering the electrode and to avoid consequent membrane clogging.

When transferred to the respiration cell, the platelet-free fractions were carefully handled to limit contact with the air. Dissolved oxygen (DO) concentrations were measured with stirring at 21.0–21.4°C and the plateau levels were recorded as the DO concentrations. Oxygen consumption was calculated by subtracting the measured DO levels of P-PRP from those of PPP and was further normalized by platelet counts. All samples were measured in quadruplicate. The number of samples tested was 15.

It is noted that according to the limitation designated by the Ethics Committee, sufficient volumes of blood samples could not be obtained for monitoring the time-course changes.

### 2.10 Statistical analysis

Data are presented as dot plots drawn using KaleidaGraph version 5 (Synergy Software, Reading, PA, United States). Horizontal bars represent the median of the data. Unless otherwise specified, one-way repeated measures analysis of variance on ranks was performed for multi-group comparisons, followed by Dunnett’s multiple comparison test (SigmaPlot 13.0; Systat Software, Inc., San Jose, CA, United States) versus individual control groups. A *P*-value < 0.05 was considered to be indicative of a statistically significant difference.

## 3 Results

**FIGURE 1:**
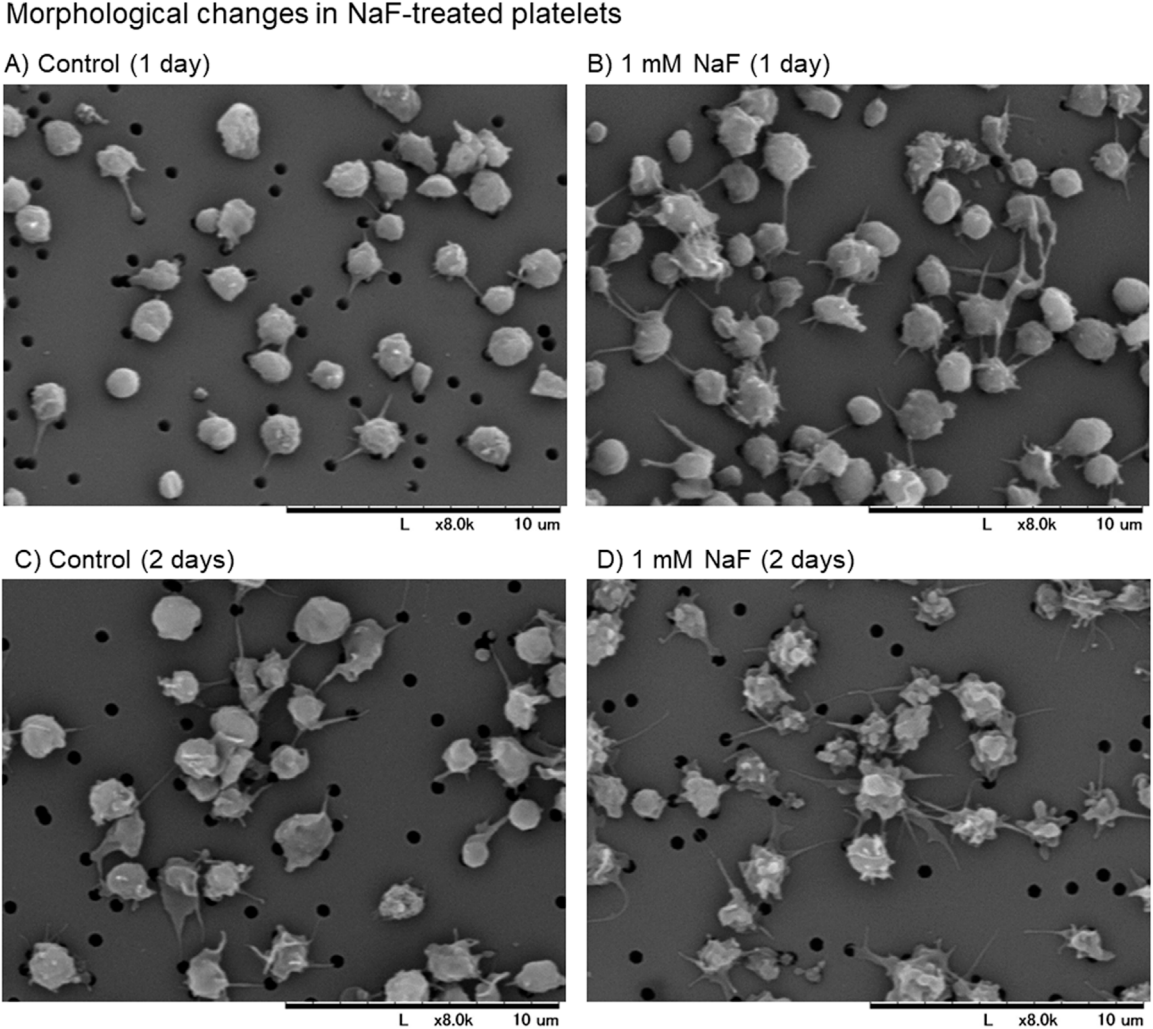
**(A–D)** The scanning electron microscopic findings of the effects of NaF on platelet appearance. Pure-platelet-rich plasma (P-PRP) was treated with NaF (1.0 mM) at ambient temperature (20°C–23°C) for 1 day **(B)** or 2 days **(D)**. The corresponding controls are shown in panels **(A, C)**. This experiment was repeated thrice, and similar observations were obtained.

The scanning electron microscopic findings of the effects of NaF on platelet appearance are shown in Figure 1AD. Under incubation conditions, the control platelets were slightly activated with time, as monitored by the expression of pseudopodia. However, platelets were significantly activated by 1 mM NaF at 1 day (Figure 1B vs A) and these morphological changes appeared more substantial at 2 days (Figure 1D vs C)Figure 1D.

**FIGURE 2:**
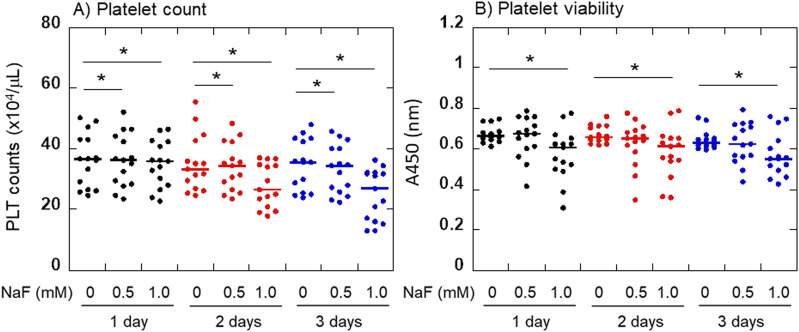
Effects of NaF on platelet viability. Platelet counts **(A)** and the converting capacities of tetrazolium salt to water-soluble formazan **(B)** were determined. For statistical analysis, Friedman repeated measures ANOVA was exceptionally applied to the comparison at 3 days in panel **(B)**. *P < 0.05, significantly different among each group.

The effects of NaF on platelet viability are shown in Figure 2. Platelet counts significantly decreased in a concentration- and time-dependent manner (Figure 2A). To confirm this finding, the number of living platelets was determined by the conversion to water-soluble tetrazolium salt (Figure 2B). Due to the additional centrifugation process, the data differed in 0.5 mM NaF from the platelet count; however, a similar tendency was observed. The significant inhibitory effects of 1 mM NaF on platelet viability were confirmed during the experiment.

**FIGURE 3:**
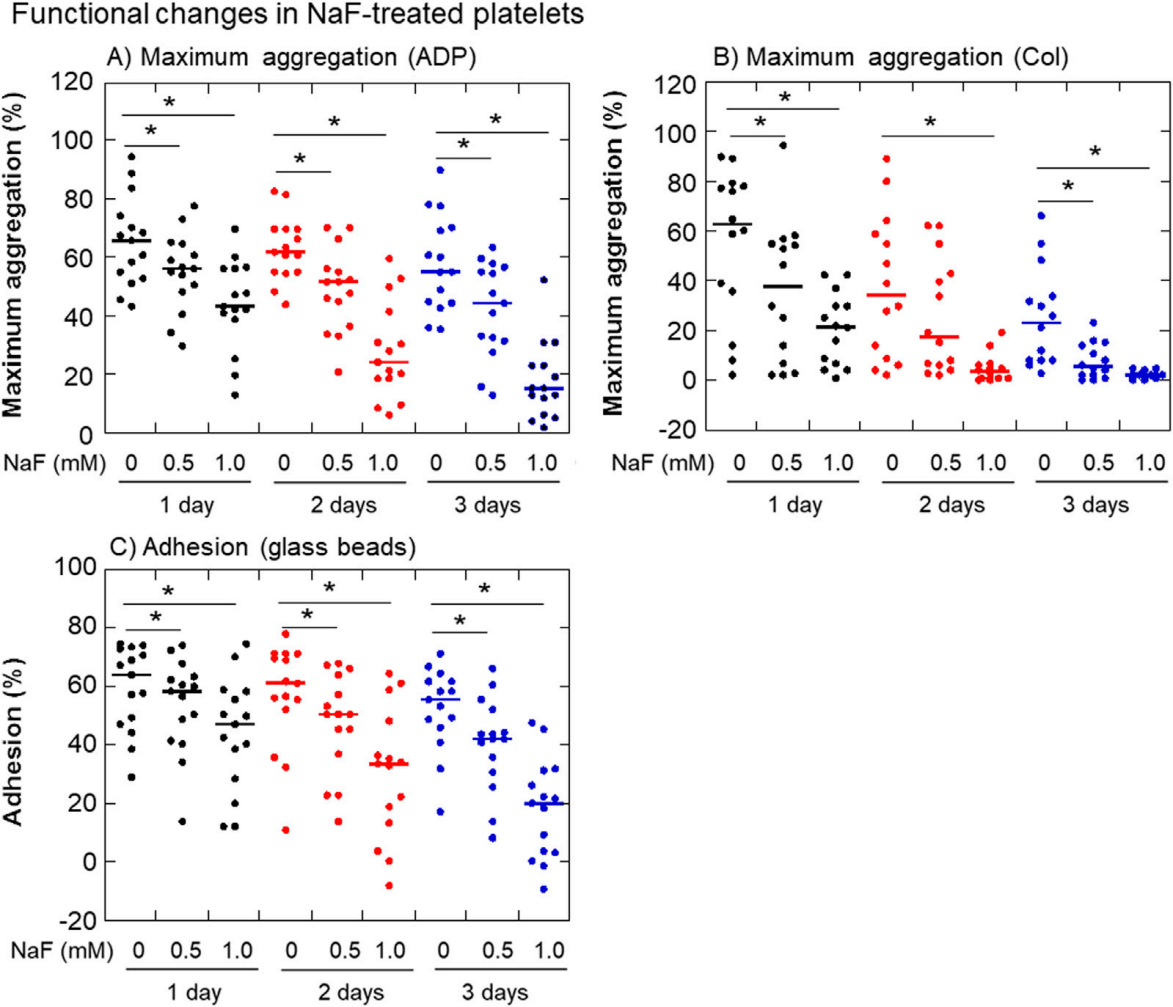
Effects of NaF on ADP- **(A)** or collagen-induced **(B)** platelet aggregation and adhesion to the glass surface **(C)**. Pure-platelet-rich plasma (P-PRP) was treated with NaF (0.5 or 1.0 mM) at ambient temperature (20–23°C) for up to 3 days. At the end of the incubation period, platelets were subjected to aggregation or adhesion assays. n = 15. *P < 0.05, significantly different among each group.

The effects of NaF on ADP- or collagen-induced platelet aggregation and adhesion to glass surfaces are shown in Figure 3. In parallel with the morphological changes, the platelet aggregation ability decreased dramatically with time. In addition, this ability seemed to vary among individual samples. Maximum platelet aggregation in response to 3 μM ADP was inhibited by NaF (0.5–1.0 mM) in a concentration- and time-dependent manner (Figure 3A). Compared with ADP, the maximum platelet aggregation in response to 2 μg/mL collagen was seemingly more complicated. However, essentially as shown in ADP, platelet aggregation in response to collagen was inhibited by NaF (0.5–1.0 mM) in a concentration- and time-dependent manner (Figure 3B). Similarly, platelet adhesion to the glass surface was significantly inhibited by NaF (0.5–1.0 mM) during treatment (Figure 3C).

**FIGURE 4:**
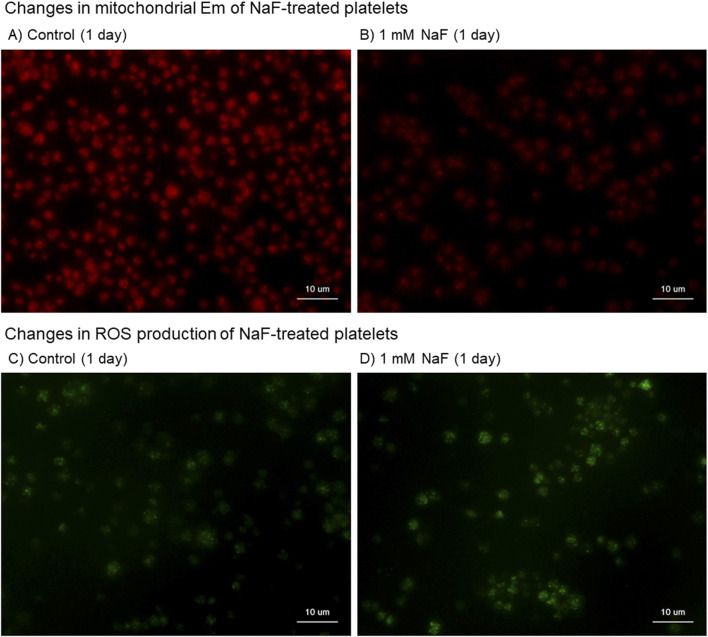
Cytochemical findings of the effects of NaF on mitochondrial membrane potential (Em) **(A, B)** and reactive oxygen (ROS) production **(C, D)** in platelets. Pure-platelet-rich plasma (P-PRP) was treated with NaF (1.0 mM) at room temperature (20°C–23°C) for 1 day. **(A, C)** Control and **(B, D)** NaF-treated platelets. This experiment was repeated thrice and similar observations were obtained.

The cytochemical findings of the effects of NaF on mitochondrial Em, which is the indicator of mitochondrial proton pump involved in OXPHOS (Zorova et al., 2018), and ROS production in platelets are shown in Figure 4. When platelets were treated with 1.0 mM NaF for 1 day, mitochondrial Em, as stained in red, substantially decreased (Figure 4B vs A). Conversely, this treatment substantially increased ROS production, as stained in green (Figure 4D vs C). In platelets treated with 0.5 mM NaF, those effects were less substantial or reproducible depending on individual blood samples.

**FIGURE 5:**
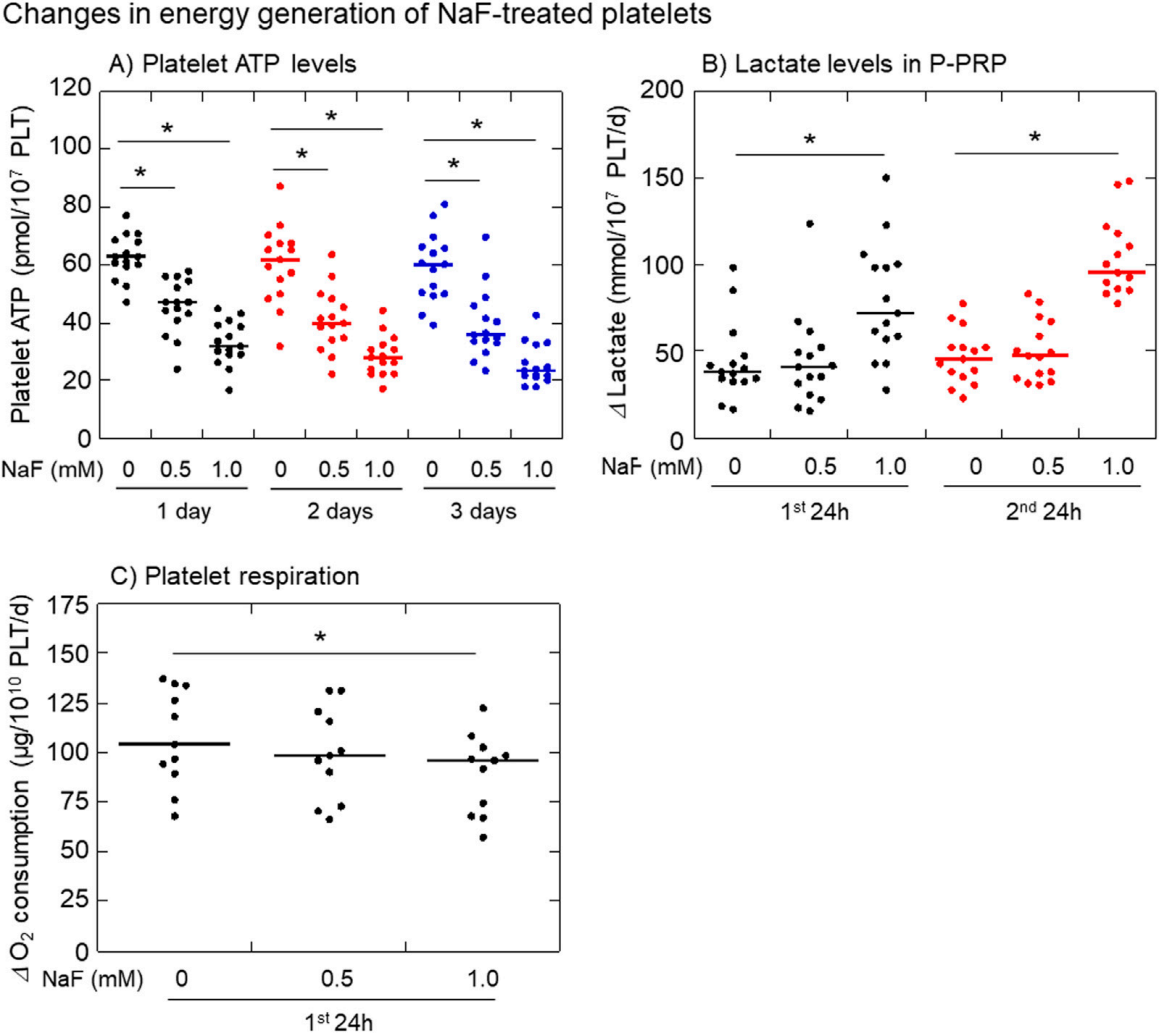
Effects of NaF on platelet ATP levels **(A)**, extra-platelet lactate levels **(B)**, and platelet oxygen consumption levels **(C)**. Pure-platelet-rich plasma (P-PRP) was treated with NaF (0.5 or 1.0 mM) at room temperature (20–23°C) for up to 3 days. Platelet ATP levels were determined at the end of each incubation period, while the changes determined both lactate and oxygen consumption levels during the first and second 24 h n = 15 **(A, B)** or 11 **(C)**. *P < 0.05, significantly different among each group.

The effects of NaF on platelet ATP levels, lactate levels in extracellular plasma, and oxygen consumption are shown in Figure 5. Platelet ATP levels were significantly decreased by NaF (0.5–1.0 mM) in a concentration-dependent manner (Figure 5A). Extra-platelet lactate levels were significantly increased by 1 mM NaF, but not 0.5 mM NaF, in a time-dependent manner (Figure 5B). Platelet oxygen consumption, which is required to the formation of proton gradient across the mitochondrial inner membrane (Bertram et al., 2006), was significantly decreased by 1 mM NaF, but not 0.5 mM NaF, during the first 24 h (Figure 5C).

## 4 Discussion

The primary question in this study was whether non-mitogenic platelets are as sensitive to reduced viability as adherent mitogenic cells to low fluoride levels. This study demonstrated that approximately 1 mM is the border of fluoride-induced cytotoxicity in human platelets, essentially as shown in many other nucleated cell types regardless of their karyotypes (Helgeland and Leirskar, 1976; Hirano and Ando, 1997; Jeng et al., 1998; He et al., 2015). In nucleated cells that have the mitogenic capacity for cell proliferation, it has been ambiguously, but generally, thought that the cytotoxic action is due to the balance of the mitogenic potential and toxic effects. The present platelet’s similarity in cytotoxic fluoride levels suggests that fluoride cytotoxic action could always be expressed universally regardless of cell mitogenic potential.

**FIGURE 6:**
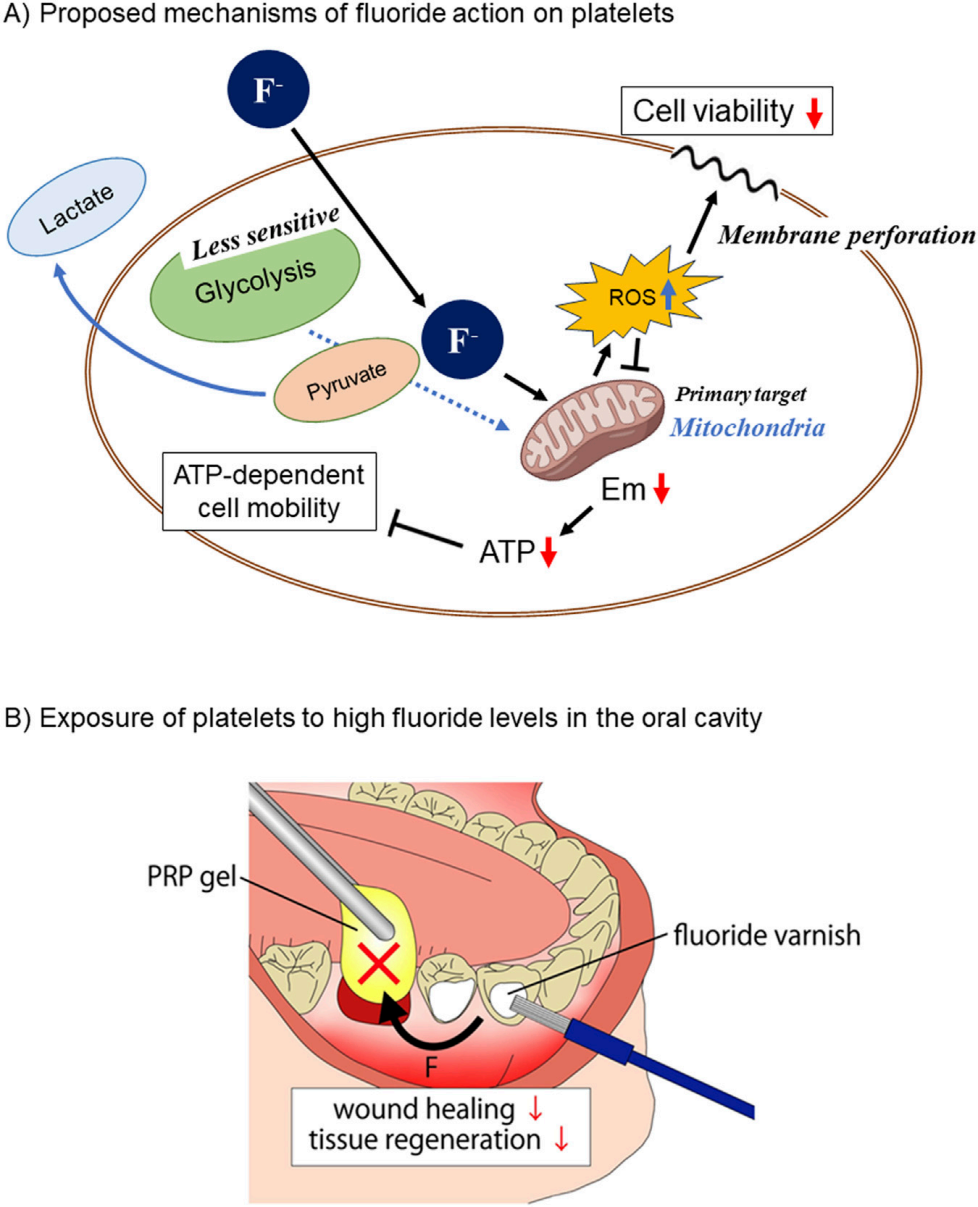
**(A)** Schematic diagram showing the proposed mechanisms of fluoride action on platelet viability, energy metabolism, and functions. Fluoride passes across the plasma membrane and primarily acts on mitochondria, resulting in decreased energy generation and increased ROS production. The former and the latter influence platelet functions and viability, respectively. **(B)** Representative clinical case of platelet exposure to high fluoride levels in the oral cavity. When wounds, such as sockets, are treated with platelet concentrates, varnishing of adjacent teeth with high fluoride levels has a high risk of impeding wound healing.

The second question was, which is the major target of fluoride involved in energy depletion and dysfunction between glycolysis and mitochondrial OXPHOS? This study also demonstrated that the fluoride-induced functional inhibition occurred simultaneously with the inhibition of mitochondrial OXPHOS as monitored by mitochondrial Em, respiration activity, and ATP level. Fluoride is well known to inhibit glycolysis (Cimasoni, 1971; ten Cate and van Loveren, 1999; Qin et al., 2006; Dimeski et al., 2015) and is thus used for laboratory testing of blood samples. Considering the general concept regarding platelet energy metabolism that the glycolysis system functions as the main ATP generator regardless of their resting or activation states (ten Cate and van Loveren, 1999; Dimeski et al., 2015; DeFronzo et al., 2016; Ryoo and Kim, 2018), it was hypothesized that fluoride primarily inhibits glycolysis to decrease extracellular lactate levels. Against this expectation, fluoride significantly increased the extra-platelet lactate levels. From a biochemical point of view, the “unexpected” changes could be explained by the concept that pyruvate is produced as usual but not efficiently utilized in the following TCA cycle and consequently accumulated and released in the form of lactate (DeFronzo et al., 2016; Ryoo and Kim, 2018). In line with this scenario, the primary target of fluoride in platelets can be considered the mitochondrial energy generation system rather than the glycolysis system (Gambino, 2013). The schematic diagram showing this mechanism is shown in Figure 6A. Judging from the dissimilarity of concentration-effectiveness relationships between viability and other indexes, it seems more likely that the reduced viability may be directly caused by increased mitochondrial ROS production rather than energy depletion because ROS induces not only mitophagy but also nonspecific damage to cell phospholipid bilayers and the plasma membrane (Cordeiro, 2014). Further investigations are required to clarify this mechanism: however, our findings regarding the positive correlations between reduced energy metabolism and dysfunction would provide an opportunity to again investigate the balance between glycolysis and OXPHOS in platelets.

The technical limitations for the determination of mitochondrial Em should be briefly discussed here. There are several fluorescent dyes, such as JC-1, tetramethylrhodamine ethyl ester (TMRE), and tetramethylrhodamine methyl ester (TMRM), to determine mitochondrial Em (Laboratories, 2024). Each dye has both advantages and disadvantages. Resistance against photobleaching is the primary critical issue for these dyes, while the secondary issue is the unavailability of fixed cells. In clinical studies using limited volumes of precious human samples, it is important to use them efficiently by performing several independent experiments in parallel, without significant loss. In line with this policy, we chose MT-1, a fluorescent dye that can be applied to fixed cells and observed after fixation (Takeda and Dai, 2022; Ushiki et al., 2022).

Concerning the limitation of this study, we should carefully interpret the *in-vitro* cell-based data. The oral cavity is always covered by biofilm that is chronically formed on its various surfaces due to the presence of rich nutrients and other favorable environments (Ray and Pattnaik, 2024) and protects against invasion of fluoride as well as mucous barrier (Bierbaumer et al., 2018). However, the biofilm is frequently washed out and reformed by saliva, drinking, and eating, whereas it could be drastically changed by periodontal and peri-implant diseases and dental caries. Thus, the situation regarding the exposure of platelets to clinically applied fluoride could vary with individual oral health conditions. It should be noted that this study examined the effects of fluoride on platelets by simply focusing on the basic contact between platelets and fluoride ions.

Compared with other tissues or organs, the oral cavity has a higher probability of exposure to high levels of fluoride. In the case of mouth rinse, NaF levels in the solution usually range from 0.05% to 0.2%, which is converted to 11.9–47.6 mM (Shimizu et al., 2022). For varnish, higher levels of NaF (2.7–5.0%; 643.0–1190.8 mM) are conventionally applied to prevent dental caries under professional control (Baik et al., 2021) (Figure 6B). These concentrations were extraordinarily higher than those used in this study. Even though the high levels of fluoride coated on the tooth surface could gradually be washed out and diluted by food, drink, saliva, and mouth-washing, biologically significant fluoride levels are expected to remain for a while and influence surrounding tissues. Thus, in the presence of wounds, cysts, or inflamed pockets in the oral cavity, it is plausible that NaF mouth rinsing or coating damages infiltrating platelets, delaying hemostasis and subsequent wound healing or exacerbating inflammation. Similar possibilities are imaginable in the case of overlapping PRP therapy. Platelets externally applied in the form of PRP could also be disrupted by the topical application of fluoride (Figure 6B); therefore, PRP may lose its efficacy in wound healing and tissue regeneration.

In the last 3 decades, PRP has been widely used for tissue regeneration in various fields including dentistry (Kawase, 2015). PRP is often used for socket preservation and alveolar bone augmentation of the oral cavity. Thus, when the fluoride coating remains on the surface of adjacent teeth, PRP’s regenerative potential of PRP could be impeded because it is not solely due to its concentrated growth factors, but also due to platelet activity (Kawase, 2022). It has been scientifically proven that fluoride, whose concentrations vary with the method of application, is beneficial for maintaining oral health through the remineralization of enamel, inhibition of bacterial growth, prevention of tooth decay, and decrease in tooth sensitivity (Pitts et al., 2017). In addition, fluoride treatment is undoubtedly a highly cost-effective therapeutic option. Therefore, this study did not indicate the overall withdrawal of fluoride from the oral cavity. Instead, clinicians should pay more attention to such topical conditions and carefully use fluoride when wounds exist nearby or when PRP is applied nearby.

## 5 Conclusion

At relatively low levels, NaF has the potential to attenuate platelet function, probably primarily through the inhibition of mitochondrial energy generation rather than glycolysis. Its cytotoxicity may be directly related to ROS production. Thus, these findings suggest that, when used topically, for example, most probably in the oral cavity, NaF could interfere with wound healing and tissue regeneration by endogenous and exogenously added platelets in the form of PRP in clinical settings.

## Data Availability

The raw data supporting the conclusions of this article will be made available by the authors, without undue reservation.
